# The unintended burden of transmission-based precautions for suspected COVID-19 in the ambulatory setting

**DOI:** 10.1017/ice.2025.10229

**Published:** 2025-09

**Authors:** Rebecca A. Stern, Katherine Bashaw, Claude E. Shackelford, Thomas R. Talbot

**Affiliations:** 1 Division of Infectious Diseases, Department of Medicine, Vanderbilt University Medical Center, Nashville, TN, USA; 2 Department of Infection Prevention, Vanderbilt University Medical Center, Nashville, TN, USA; 3 Department of Medicine, Vanderbilt University Medical Center, Nashville, TN, USA

## Abstract

An observational pilot in walk-in clinics assessed workflow impacts of personal protective equipment (PPE) use for COVID-19 cases. PPE added time, waste, and cost despite a low incidence period of illness. Limited supporting data for contact transmission and operational barriers suggest ambulatory PPE guidance for COVID-19 warrants modification.

Current guidelines for suspected or confirmed COVID-19 cases in any healthcare setting recommend airborne and contact precautions with eye protection (N-95 mask, gown and gloves, face shield or goggles).^1^ The use of transmission-based precautions, particularly for COVID-19, has emerged largely from experiences in inpatient settings, with sparse insights on application of these precautions in ambulatory clinics. In ambulatory settings, there may be more opportunities for immediate source control and reduced risk of recurrent environmental contamination of pathogens due to the shorter time spent in the healthcare setting by patients. The challenges introduced with blanket implementation of transmission-based precautions in ambulatory settings, particularly regarding workflow, capacity strains, and supply limitations, must be assessed to allow for reliable use of these precautions to protect patients and healthcare personnel (HCP).

The role of contact transmission for SARS-CoV-2 has been specifically questioned, noting the major route of transmitting SARS-CoV-2 virus is airborne.^2–5^ This timed analysis of the currently recommended isolation precautions for suspected COVID-19 in walk-in clinics (WIC) aimed to identify unintended impacts of these precautions.

## Methods

An observational pilot was conducted across four WICs in an academic hospital network from July to October 2024. Patients who screened positive at check-in with cough, sore throat, congestion, or recent COVID-19 positive testing triggered an electronic notification on the need for airborne and contact isolation precautions with eye protection. A timed evaluation of HCP personal protective equipment (PPE) donning and doffing at patient room entry and exit was performed by two infection prevention (IP) team members using a standardized process with a stopwatch. Times were reported to don and doff PPE per instance of entering and exiting a room by a single provider. Individual room entries and exits for the same patient, whether by the same or different HCP, were recorded. Added time, resource utilization (PPE units) and cost were extrapolated to monthly and annual totals for WICs to assess operational and workflow impacts in low and high incidence periods of respiratory viral illness (RVI).

Cost analysis was performed using a per unit value of $3.95 for each PPE unit based on average supply component pricing at the time of the pilot (includes N-95 mask, gown, gloves, face shield). Financial impact comparisons for PPE were made in low versus high incidence periods of RVI, using 30% as observed during the pilot data collection period (low) and 50% as an estimated high projection, assuming stable clinic volumes.

## Results

Sixty patient encounters were observed, representing 30.4% of the total WIC patients seen during the observation periods (N = 197 over 36.5 hours; Table 1). The mean time to don and doff PPE per room entry and exit was 1.58 and 0.57 minutes, respectively (2.15 minutes per don and doff cycle; Table 2). HCP performed donning and doffing an average of 1.8 times (range 1–4) per patient. HCP time assessments may have included a single HCP donning and doffing multiple times or several HCP donning and doffing for the same patient encounter. This added 3.9 minutes per patient encounter (or per HCP per encounter) requiring PPE. Extrapolated to a 12-hour shift (using clinic patient volume averages from January 2021 to October 2024), this added 1.3 hours to daily activities and encompassed 35 sets of PPE (eg, gowns, gloves, eye protection, respirators), contributing to WIC waste volumes. Calculations extrapolating to all 12 WICs, if assuming 30% of patients would require isolation based on pilot data with >18,000 total WIC visits/month, translated monthly to an additional 355 hours of donning/doffing time, 9,835 PPE units used, and PPE cost of $38,849.

**Table 1.** Cohort description at selected WICs

WIC	All pts seen during observation period	No. (%) WIC pts requiring isolation	Symptom/condition triggering isolation
A	72	21 (29.1%)	Cough – 33 (55%) Congestion – 13 (21.7%) Sore throat – 33 (55%) COVID-19 – 6 (10%)
B	45	11 (24.4%)	
C	20	8 (40%)	
D	60	20 (33.3%)	
Overall	197	60 (30.4%)	

**Table 2.** Use of PPE for potential or confirmed COVID-19 cases in WICs

WIC	Pts requiring isolation	Avg. time to don PPE (min)	Avg. time to doff PPE (min)	Avg. time to don and doff PPE (min)	Don/Doff events total	Avg. number of times PPE donned and doffed per pt
A	21	1.62	0.75	2.38	37	1.76
B	11	1.23	0.37	1.60	16	1.45
C	8	1.49	0.42	1.91	25	3.13
D	20	1.81	0.58	2.39	30	1.50
Overall	60	1.58	0.57	2.15	108	1.80

PPE, personal protective equipment.

## Discussion

Current COVID-19 PPE requirements increased burdens to WIC workflows including added time, high resource utilization, and excess environmental waste. Sentiments by frontline HCP reinforced these results during the pilot (results from an accompanying voluntary survey by these researchers), indicating a majority strong agreement that PPE increased the time required, burden to HCP, and waste during patient encounters.^6^ These challenges reduce the likelihood of HCP wearing PPE in ambulatory settings when encountering communicable diseases, as seen with frontline HCP at Colorado primary and urgent care clinics where only 4% of HCP exposed to Mpox cases adhered to all recommended PPE.^7^

Notably, the pilot occurred during a period of relatively low community incidence of COVID-19 and influenza, meaning that projections for workflow effects, capacity reductions, PPE waste, and cost would be substantially higher during expected peak periods of illness. These data likely underestimate the true amount of time added, as the mean number of 1.8 donning and doffing cases per patient appears lower than expected for a typical WIC encounter. Process mapping of clinic capacity and logistics are essential to understand how current PPE requirement impacts factor into each touchpoint of clinic, including triage/intake and provider examination.

Transmission-based precautions for suspected or confirmed COVID-19 in the ambulatory setting should be modified to remove the contact requirement of isolation (gown and gloves) given a paucity of data to support its use, limited risk of transmission by non-airborne routes, ability to promptly identify suspected patients and apply source control measures such as recommending patients to wear a mask if symptomatic, and environmental harms.^8–10^ This underscores the importance of screening for highly transmissible pathogens and optimizing proper respiratory PPE use rather than inefficient use of resources. In addition, the risk to HCP in ambulatory clinic settings is deemed very low given the short duration of clinic encounters (which would markedly limit environmental contamination with SARS-CoV-2 virus, along with cleaning high touch surfaces between patient encounters) and lack of performance of aerosol-generating procedures in most areas. Some healthcare systems have de-escalated the contact component of COVID-19 PPE guidelines without a noted signal of harm for HCP or patient safety and have benefited from reduced environmental waste and cost.^11^

These proposed PPE modifications may improve clinic efficiency and decrease burdens to HCP. Successful transitions in healthcare systems would benefit from making PPE easily available to all HCP for voluntary use if desired, though gown and gloves would not be required. Emphasis on patient and HCP masking and the use of standard precautions should continue.

Potential limitations of this study include the assumption that time to don and doff PPE would remain static over time when extrapolating data, though practically HCP would be anticipated to become more adept and faster with experience. A small subset of observations noted improper donning and doffing techniques, such as keeping masks on after leaving the room, which likely shortened the time recorded for PPE. The IP team provided real-time education to correct those gaps. Noncompliance with PPE by a smaller subset of HCP was noted though not formally tracked for this pilot. Possible implementation generalizability limitations of these data involve resource availability at WICs outside this pilot, including underserved areas, which are not uniform and may pose barriers to compliance with current national guidance.

De-escalation of PPE requirements for ambulatory care of patients with suspected or confirmed COVID-19 is worthwhile to pursue to promote safe, efficient, and durable practices. Challenges of scale and practicality must be balanced with risk and resiliency, particularly as the ambulatory care footprint expands. Current COVID-19 PPE guidance merits a change to the contact component given limited benefit both scientifically and operationally, harms of isolation gowns, and opportunity to safely transition to a new standard.
